# Identification of circulating metabolites associated with wooden breast and white striping

**DOI:** 10.1371/journal.pone.0274208

**Published:** 2022-09-26

**Authors:** Juniper A. Lake, Yiren Yan, Jack C. M. Dekkers, Jing Qiu, Erin M. Brannick, Behnam Abasht

**Affiliations:** 1 Center for Bioinformatics and Computational Biology, University of Delaware, Newark, Delaware, United States of America; 2 Department of Animal and Food Sciences, University of Delaware, Newark, Delaware, United States of America; 3 Institute for Financial Services Analytics, University of Delaware, Newark, Delaware, United States of America; 4 Department of Animal Science, Iowa State University, Ames, Iowa, United States of America; 5 Department of Applied Economics and Statistics, University of Delaware, Newark, Delaware, United States of America; Cairo University, EGYPT

## Abstract

Current diagnostic methods for wooden breast and white striping, common breast muscle myopathies of modern commercial broiler chickens, rely on subjective examinations of the pectoralis major muscle, time-consuming microscopy, or expensive imaging technologies. Further research on these disorders would benefit from more quantitative and objective measures of disease severity that can be used in live birds. To this end, we utilized untargeted metabolomics alongside two statistical approaches to evaluate plasma metabolites associated with wooden breast and white striping in 250 male commercial broiler chickens. First, mixed linear modeling was employed to identify metabolites with a significant association with these muscle disorders and found 98 metabolites associated with wooden breast and 44 metabolites associated with white striping (q-value < 0.05). Second, a support vector machine was constructed using stepwise feature selection to determine the smallest subset of metabolites with the highest categorization accuracy for wooden breast. The final support vector machine achieved 94% accuracy using only 6 metabolites. The metabolite 3-methylhistidine, which is often used as an index of myofibrillar breakdown in skeletal muscle, was the top metabolite for both wooden breast and white striping in our mixed linear model and was also the metabolite with highest marginal prediction accuracy (82%) for wooden breast in our support vector machine. Overall, this study identified a candidate set of metabolites for an objective measure of wooden breast or white striping severity in live birds and expanded our understanding of these muscle disorders.

## Introduction

Wooden breast and white striping are breast muscle disorders of modern commercial broiler chickens that inflict a substantial economic burden on the poultry industry worldwide due to the high prevalence and detrimental effects of these diseases on meat quality and appearance. Despite distinct appearances by gross analysis, these myopathies are believed to be part of the same disease spectrum or progression [1,2], frequently manifest together in the same bird, and have high genetic correlation with each other (r = 0.9) [3]. At the microscopic level, both disorders present with similar lesions, including myodegeneration with regeneration, necrosis, lymphocyte and macrophage infiltration, fibrosis, and lipidosis [4,5]. However, severe wooden breast is clinically and grossly characterized by palpable firmness of the pectoralis major muscle, particularly at the cranial end, while white striping appears grossly as fatty white striations that run parallel to the muscle fibers.

Although the exact etiologies of these myopathies are still not fully understood, it is clear that both genotype and environmental factors play a role [3,6]. One hypothesis proposes shared pathogenesis with metabolic syndrome and type 2 diabetes mellitus in mammals, wherein differences in insulin signaling and glucose transport in the skeletal muscle of chickens produce symptoms most akin to mammalian diabetic complications in the heart, liver, and kidneys [7]. Other hypotheses implicate rapid growth of the pectoralis major muscle relative to its surrounding epimysium, which is believed to marginalize vasculature and thus impair supply of oxygen and nutrients, inhibit removal of metabolic waste, and eventually precipitate the degeneration of muscle fibers and abnormal remodeling of the extracellular matrix [1,4,8]. However, further research into the biological basis of wooden breast and white striping is hampered by current diagnostic techniques, which rely primarily on manual palpation and visual examination of the breast muscle. Such techniques are either not very accurate in live birds (palpation) or not applicable to live birds (gross examination of muscle at processing or necropsy) and can be biased by the subjectivity and inconsistency between scorers. Diagnosis using histology or muscle metabolomics can be performed on live birds using muscle biopsies [9,10], but this technique is invasive, substantially affected by sampling site, and not well suited to studies that require repeated measurements over time. A diagnostic model based on blood, plasma, or serum metabolites would allow for objective and repeatable quantification of disease severity in live birds, thus vastly improving the quality of diagnostics and research relating to wooden breast and white striping.

Metabolomic approaches have previously been used to identify metabolites and key metabolic pathways associated with wooden breast and white striping in broiler chickens. For example, Abasht et al. [11] highlighted altered lipid and carbohydrate metabolism in birds affected with wooden breast, as well as changes in histidine and glutathione metabolism that may indicate increased inflammation, oxidative stress, and muscle protein breakdown. Similarly, Boerboom et al. [12] found increased levels of long-chain fatty acids and signs of perturbations to the citric acid cycle associated with white striping. However, it has become evident that the effects of such breast muscle myopathies extend beyond the pectoralis major muscle, with systemic changes described in the vasculature, blood, lungs, and other skeletal muscles [4,13–15]. One such systemic change associated with wooden breast and white striping is increased creatine kinase activity in serum, which is a known marker of muscle damage or degeneration [16,17].

As previous metabolomics studies have generally been limited to the pectoralis major muscle or target only a few proteins, more comprehensive metabolomic profiling of wooden breast and white striping using blood can provide novel insights about the diseases from a more systemic perspective. In this study, we applied an untargeted metabolomics approach to identify plasma metabolites associated with wooden breast and white striping in male broiler chickens at market age. The primary objectives of this study were (i) to identify plasma metabolites that can be used to identify and objectively quantify wooden breast and white striping in live birds and (ii) to provide insights into the underlying pathophysiology of these myopathies.

## Materials and methods

### Test animals, study design, and sampling

All animal procedures were performed in accordance with guidelines set by The University of Delaware Institutional Animal Care and Use Committee (IACUC) and were approved by IACUC under protocol number 48R-2015-0. The sample population consisted of 250 commercial broiler chickens selected from a previously described study population [3] according to wooden breast scores and sex determined at necropsy (selection criteria described below). All birds were offspring from the same breeding population of 15 sires and 200 dams, but were raised in two separate hatches (n1 = 109, n2 = 141). Broilers were housed according to optimal industry standards in five poultry houses on the University of Delaware Newark campus farm complex (Newark, DE) and given free access to feed and water until approximately 7 weeks of age, at which time they were euthanized by cervical dislocation. Due to the complexity of standardized sample collection across a large number of birds, bird necropsy was conducted over 4 days, at 48, 49, 52, and 53 days of age, once the birds had reached full market weight. Preceding euthanasia, live weight was recorded and whole blood samples were collected from the brachial wing vein of each bird using a 3mL syringe with 23-gauge needle and placed in lithium heparin-coated tubes. Plasma was separated by centrifugation and stored at -80˚C until further analysis. During necropsy, the pectoralis major muscles were evaluated visually and by manual palpation for gross lesions and palpable firmness associated with wooden breast. Each bird was assigned a wooden breast score according to the 5-point scale described by Lake et al. [3], with the exception that "1-Very Mild" was renamed "1-Minimal": 0-Normal, 1-Minimal, 2-Mild, 3-Moderate, and 4-Severe. White striping was also assessed at this time and each bird was assigned a white striping score using a 4-point scale described by Lake et al. [3]: 0-Normal, 1-Mild, 2-Moderate, and 3-Severe.

Plasma samples from 250 birds were selected for metabolomics profiling using the following criteria. First, birds associated with plasma samples with volumes less than 120 μl were not included. Second, birds were filtered to retain only those determined to be males at necropsy and otherwise in good health (i.e. no indication of ascites, leg defects, or other disease conditions beyond wooden breast or white striping). Third, birds were selected based on wooden breast score to maximize statistical power in detecting differences between the extremes of the wooden breast disease spectrum. Samples from birds receiving extreme scores (0-Normal, 3-Moderate, and 4-Severe) were selected first and then additional samples were randomly selected from the pool of birds with scores of 1-Minimal. No birds with scores of 2-Mild were included in this study. Sex was later confirmed using genetic analysis of sex chromosomes [3].

### Metabolomic sample Accessioning and preparation

Frozen plasma samples were thawed at room temperature and 120 μl aliquots of each sample were placed in individual 2.0 mL polypropylene tubes before being immediately flash frozen in liquid nitrogen. Frozen aliquots were shipped on dry-ice to Metabolon Inc. (Durham, NC) for metabolomics profiling using ultrahigh performance liquid chromatography-tandem mass spectrometry (UPLC-MS/MS).

Following receipt, samples were inventoried and immediately stored at -80˚C until processing. Each sample received was accessioned into the Metabolon Laboratory Information Management System (LIMS) system and was assigned a unique identifier that was used to track all sample handling, tasks, and results. The samples (and all derived aliquots) were tracked by the LIMS system.

Samples were prepared using the automated MicroLab STAR system (Hamilton Company). Several recovery standards were added prior to the first step in the extraction process for quality control purposes. To remove protein, to dissociate small molecules that were bound to protein or trapped in the precipitated protein matrix, and to recover chemically diverse metabolites, proteins were precipitated with methanol under vigorous shaking for 2 min (Glen Mills GenoGrinder 2000), followed by centrifugation. The resulting extract was divided into five fractions for analysis using four different methods, and one sample reserved for backup. Samples were placed briefly on a TurboVap (Zymark) to remove the organic solvent and then stored overnight under nitrogen before preparation for analysis. Several types of controls were analyzed in concert with the experimental samples; a detailed description of the quality control methods can be found in S1 File.

### Ultrahigh performance liquid chromatography-tandem mass spectroscopy (UPLC-MS/MS)

All mass spectroscopy methods utilized a Waters ACQUITY ultra-performance liquid chromatography (UPLC) and a Thermo Scientific Q-Exactive high resolution/accurate mass spectrometer interfaced with a heated electrospray ionization (HESI-II) source and Orbitrap mass analyzer operated at 35,000 mass resolution. The sample extract was dried then reconstituted in solvents compatible to each of the four methods: two separate reverse phase (RP)/UPLC-MS/MS methods with positive ion mode electrospray ionization (ESI), one for analysis by RP/UPLC-MS/MS with negative ion mode ESI, one for analysis by hydrophilic interaction liquid chromatography (HILIC)/UPLC-MS/MS with negative ion mode ESI. The scan range varied slighted between methods but covered 70–1000 m/z.

Samples were balanced across multiple run days to conserve the ratio of wooden breast scores and to help account for inter-day tuning differences in instruments. Additional details on the UPLC-MS/MS methods are available in S1 File.

### Compound identification and quantification

Metabolites were identified and quantified by Metabolon using Metabolon’s hardware and software. Compounds were identified by comparison to library entries of purified standards or recurrent unknown entities. Biochemical identification was based on three criteria: retention time within a narrow refractive index window of the proposed identification, accurate mass match to the library +/- 10 ppm, and the MS/MS forward and reverse scores between the sample data and authentic standards. The size of peaks was quantified using area-under-the-curve.

### Data pre-processing

To account for inter-day tuning differences of instruments, area-under-the curve values for each metabolite were divided by the median value of their associated run-day to equalize the medians across run-day. Metabolites or samples that contained more than 20% missing values were removed and the remaining values were log transformed and standardized to set the mean of each metabolite equal to zero and the standard deviation equal to one. Missing values, which were primarily associated with lower limits of detection, were imputed using the minimum value for that metabolite. All subsequent analyses were performed using these log-transformed, standardized and imputed metabolite values.

### Statistical analysis

Metabolites that passed the filter criteria described above were individually tested for a relationship with wooden breast score using the following linear mixed model implemented with the ‘lme4qtl’ package version 0.2.2 [18] in R:

y=Xb+Zu+e, (1)

where ***y*** is a vector of standardized metabolite values, ***b*** is the vector of fixed effects and the overall mean (a vector of 1’s), ***X*** an incidence matrix for fixed effects, ***u*** is a vector of random polygenic effects, ***Z*** is an incidence matrix corresponding to ***u***, and *e* is the residual error. Fixed effects included wooden breast score (or white striping score) and poultry house as discrete class variables and body weight at 7 weeks as a continuous variable. The effects of hatch and age at blood sample collection were both fully nested (collinear) with poultry house and, therefore, not included in the model. Random effects *u* and *e* were assumed to follow normal distributions: $u∼N(0,\sigma_{g}^{2}G)$ and $e∼N(0,\sigma_{e}^{2}I_{n})$, where $\sigma_{g}^{2}$ is the genetic variance, $\sigma_{e}^{2}$ is the variance of the residual errors, ***G*** is the genomic relationship matrix acquired from Lake et al. [3], and ***I***_*n*_ is an identity matrix of dimension *n*. Since the ‘lme4qtl’ package does not provide estimates of significance, a type II Wald chi-square test was performed with the R package ‘car’ version 3.0–10 [19] to test the significance of wooden breast or white striping score for each metabolite. Metabolites were considered to have a significant association with wooden breast or white striping score if the FDR-adjusted p-value, also referred to as q-value, was below 0.05 [20]. Pairwise comparisons of the estimated marginal means for each wooden breast and white striping score were performed for significant metabolites with the R package “emmeans” [21]. Pathway enrichment analysis of significant metabolites associated with wooden breast was conducted with MetaboAnalyst 5.0 (https://www.metaboanalyst.ca) using the *Gallus gallus* KEGG pathway library, with significance assessed using a hypergeometric test.

To assess the usefulness of metabolic traits in genetic studies of wooden breast, heritability was calculated for each metabolite as the ratio of genetic to phenotypic variance, i.e. the sum of genetic and residual variance, using genotype data from Lake et al. [3]. Heritability estimation was performed with GCTA version 1.26.0 using the same model described above, except that fixed effects only included poultry house and body weight. Results are reported only for significant metabolites.

### Construction of support vector machine (SVM) classifier

Support vector machine (SVM) is a supervised machine learning algorithm that is frequently used for classification and dimensionality reduction, especially in instances where data may not be normally distributed or may contain substantial outliers. It maps labeled training samples to points in space and selects a decision boundary between categories so as to maximize the distance between that boundary and the data points in each category [22]. Test samples are then mapped into that same space and predicted to belong to a category based on which side of the boundary they fall. To improve this model’s classification power, birds with wooden breast scores of 0-Normal and 1-Minimal were consolidated into a single group that will be called “Unaffected” and birds with wooden breast scores of 3-Moderate and 4-Severe were consolidated into a single group that will be called “Affected”.

The SVM with the optimal subset of metabolites for classifying birds as wooden breast Affected or Unaffected was constructed using an integrated machine learning feature selection algorithm, implemented in the R package ‘caret’ version 3.6.3 [23]. Specifically, stepwise selection was implemented to determine the smallest subset of metabolites with the highest prediction accuracy (proportion of true positives). First, the metabolite with the highest prediction accuracy calculated through leave-one-out cross-validation was added to the SVM. At each subsequent step, a new metabolite was added or an existing metabolite was removed if the prediction accuracy was increased according to leave-one-out cross-validation. The stepwise selection was complete when adding metabolites to the SVM or removing them from the SVM failed to increase prediction accuracy. All metabolites included in the final SVM were considered important for prediction with their relative importance concordant with the order in which they were added to the SVM.

Although radial and linear kernels were both evaluated, the linear kernel produced higher accuracy and is reported here with its only hyperparameter, cost (*C*), which is a misclassification penalty that was tuned to achieve the highest average prediction accuracy by nested cross-validation. The final SVM had 6 metabolite predictors and a *C* of 0.1.

## Results

### Quality control

A total of 615 metabolites were detected and quantitated (S1 Table), with 581 compounds passing quality control filter criteria. Of the 250 birds from that were sampled for metabolomic profiling, 4 were found to be genetically female despite being recorded as male at necropsy and were excluded from all subsequent analyses. The distributions of wooden breast score and white striping score among the remaining 246 male broilers are described in Table 1. One bird with a wooden breast score of 3-Moderate and a white striping score of 2-Moderate did not pass sequencing quality control and was excluded from the linear mixed model analysis, which controlled for relatedness by means of a genomic relatedness matrix constructed from SNP genotype data [3].

**Table 1:** Distribution of wooden breast and white striping scores among 7-week-old broiler chickens sampled for metabolomic analysis and confirmed to be genetically male (n = 246 total).

	**Wooden Breast Score**				
	0-Unaffected	1-Minimal	2-Mild	3-Moderate	4-Severe
n	68	23	0	135	20
	**White Striping Score**				
	0-Unaffected	1-Mild	2-Moderate	3-Severe	
n	59	79	82	25	

### Plasma metabolites associated with wooden breast and white striping

**Fig 1:**
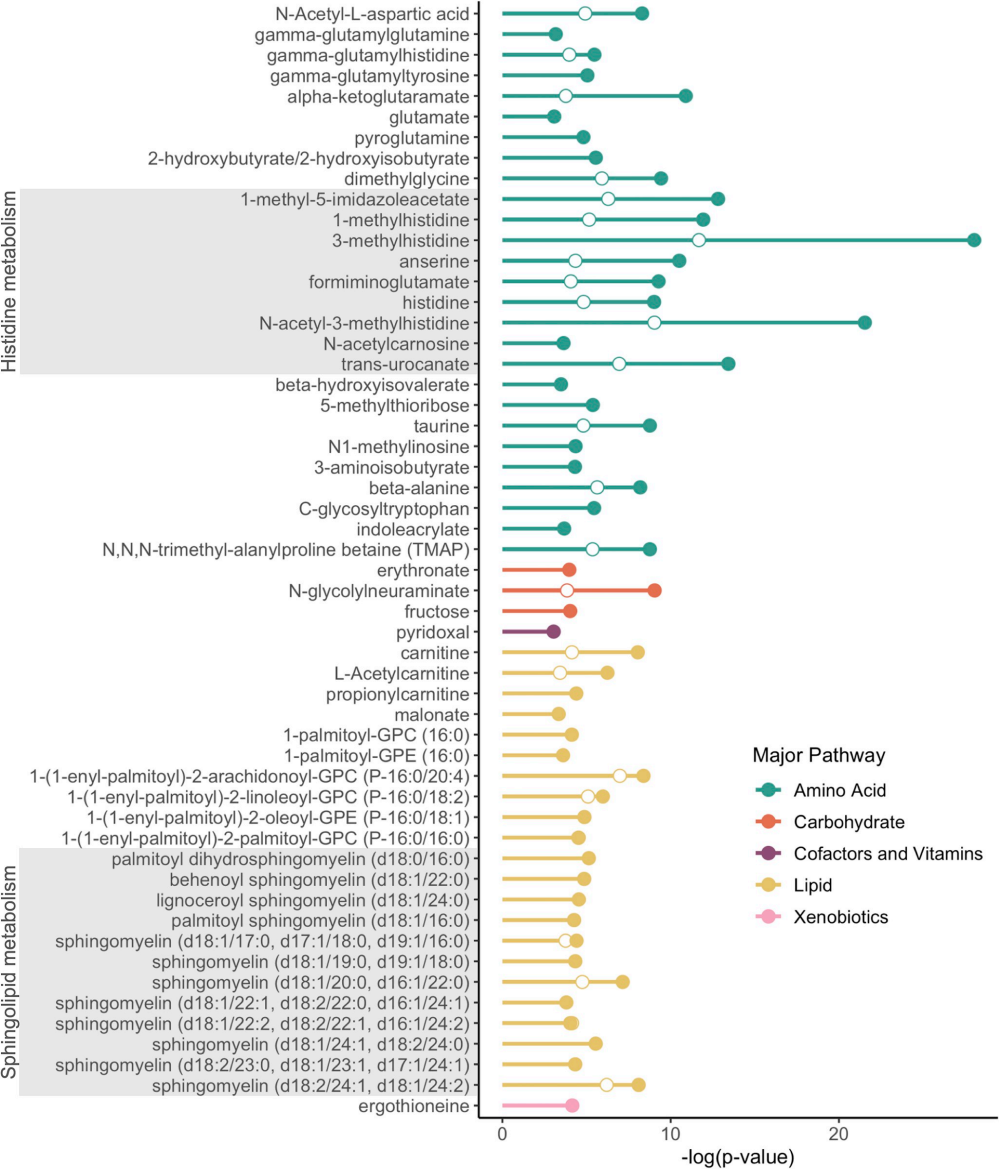
Metabolites associated with wooden breast and white striping. Top metabolites (q-value < 0.01) associated with wooden breast (closed circles) and white striping (open circles) in male broiler chickens at market age (7 weeks).

The association of wooden breast and white striping scores with metabolite levels was first investigated using a mixed linear model, which identified 98 metabolites that were significantly associated with wooden breast score and 44 metabolites that were significantly associated with white striping score (S2 and S3 Tablees). The metabolites that were associated with white striping score were almost entirely a subset of those found to be significant for wooden breast, with only 4 metabolites that were unique to white striping– 1-palmitoyl-2-arachidonoyl-GPE (16:0/20:4), 1-palmitoyl-2-oleoyl-GPE (16:0/18:1), chiro-inositol, and stearoyl sphingomyelin (d18:1/18:0). The majority of significant metabolites were amino acids or lipids, with the greatest effects of wooden breast and white striping relating to histidine metabolism and sphingolipid metabolism (Fig 1). The top metabolite for both muscle disorders was 3-methylhistidine, which showed clear differences between birds with no apparent disease and those with even minimal disease (S2 and S3 Tables).

### Pathway enrichment

A total 75 of the 98 metabolites identified as significant for wooden breast were recognized by the MetaboAnalyst database. Pathway enrichment analysis found two significant pathways–histidine metabolism and beta-alanine metabolism. The results of this analysis, including the metabolites associated with each significant pathway, are presented in Table 2.

**Table 2:** Results of pathway enrichment analysis for wooden breast metabolites.

Pathway	FDR	Metabolites
Histidine metabolism	0.000016	3-methylhistidine; anserine; glutamate; histamine; histidine; formiminoglutamate; trans-urocanate
Beta-alanine metabolism	0.073	5,6-dihydrouracil; anserine; beta-alanine, histidine, spermine

### Metabolite heritability

Genetic marker data from Lake et al. [3] was used to estimate heritability for metabolites significantly associated with wooden breast score. Metabolite heritability estimates varied widely (S4 Table), with several metabolites including histamine and pyroglutamine showing no heritability (h^2^ = 0) and one metabolite, glutarylcarnitine (C5-DC), showing the highest heritability (h^2^ = 1). Notably, the top metabolite associated with both wooden breast and white striping in this study, 3-methylhistidine, showed high heritability (h^2^ = 0.90 ± 0.18). Although heritability estimates can be unique to a population due to homogeneity of the environment and genetic background, these results support a strong genetic component to plasma 3-methyhistidine levels that could respond to breeding. Additional research would be necessary to explore potential non-target effects of such marker-assisted breeding (i.e. phenotypic changes not related to wooden breast and white striping severity) and confirm its effectiveness at reducing myopathy.

### SVM classification

The optimized SVM achieved prediction accuracy of 94.3% using the leave-one-out cross-validation method with the following six metabolite predictors: 3-methylhistidine, N-acetyl-L-aspartic acid, glycerate, N,N,N-trimethyl-5-aminovalerate, alanine, and O-sulfo-L-tyrosine. The marginal prediction accuracy associated with each metabolite in the model is reported in Table 3. The full confusion matrices reporting the number of true positives, true negatives, false positives, and false negatives for each iteration can be found in S5 Table.

**Table 3:** Optimal metabolite set for wooden breast classification using support vector machine with stepwise feature selection and relevant performance measures. Accuracy is calculated as true positives / (true positives + false positives + true negatives + false negatives). Marginal prediction accuracy is the increase in total prediction accuracy achieved by adding the associated metabolite. Precision is calculated as true positives / (true positives + false positives). Recall is calculated as true positives / (true positives + false negatives). F_1_ score is calculated as 2 × (precision × recall)/ (precision + recall).

Order	Metabolite	Marginal Accuracy	Total Accuracy	Precision	Recall	F_1_ Score
1	3-methylhistidine	0.817	0.817	0.844	0.871	0.857
2	N-acetyl-L-aspartic acid	0.073	0.890	0.900	0.929	0.914
3	glycerate	0.017	0.907	0.913	0.942	0.927
4	N,N,N-trimethyl-5-aminovalerate	0.020	0.927	0.925	0.955	0.940
5	alanine	0.012	0.939	0.938	0.968	0.952
6	O-sulfo-L-tyrosine	0.004	0.943	0.938	0.974	0.956

**Fig 2:**
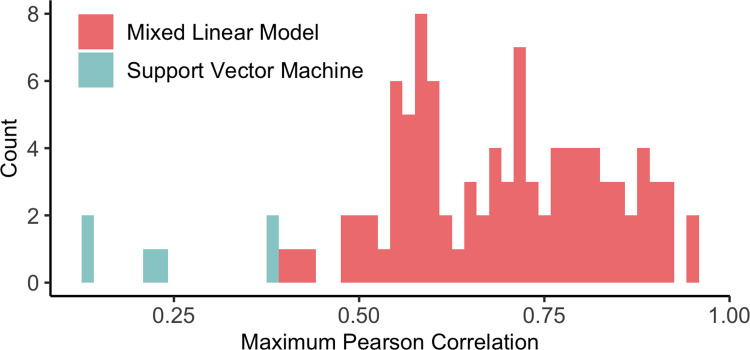
Correlation of metabolites associated with wooden breast. Maximum pairwise correlation among the 98 significant metabolites in the wooden breast mixed linear model analysis (pink) and among the 6 metabolites included in our final support vector machine (blue).

Only two of the metabolites in our final SVM, 3-methylhistidine and N-acetyl-L-aspartic acid, were identified as significantly associated with wooden breast in the previously described mixed linear model analysis. The relatively low overlap of results between these two analyses is likely due, at least in part, to correlation structure among metabolites, which is considered redundant information with regards to prediction accuracy of a classification model. We investigated this idea by calculating the Pearson correlation coefficient (*r*) for each pair of the 98 significant metabolites from the wooden breast mixed linear model analysis and each pair of the 6 metabolites included in our final SVM. Then, we plotted the maximum *r* for each of those metabolites (Fig 2) and found that maximum pairwise correlations were lower among the 6 SVM metabolites than among the 98 metabolites identified through mixed linear modeling.

## Discussion

A major finding of this study is that the metabolites associated with white striping are almost entirely a subset of those associated with wooden breast. This supports the idea that white striping may be a less severe form of wooden breast or perhaps an earlier stage of the same disease, as suggested by Griffin et al. [1]. Based on this overlap, the major findings of this study are discussed according to metabolic groups rather than divided by wooden breast and white striping.

### Histidine metabolism

**Fig 3:**
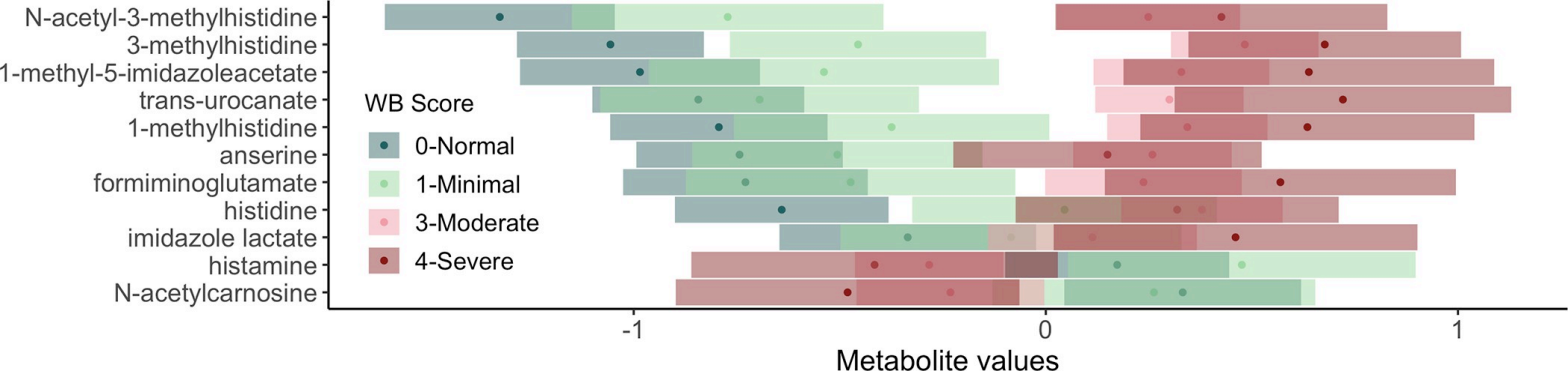
Association of wooden breast score with histidine metabolism in plasma of male broiler chickens. Results of post-hoc analysis of metabolite values by wooden breast score are shown with estimated marginal means (dots) and confidence intervals (bars). Overlapping confidence intervals indicate that there is no significant difference between the two scores. To improve visualization, metabolites are listed in order of the adjusted means for a wooden breast score of 0-Normal.

Histidine and eight additional metabolites related to histidine metabolism was elevated in association with wooden breast score (Fig 3) and white striping score. These metabolites include 1-methylhistidine, 1-methyl-5-imidazoleacetate, 3-methylhistidine, anserine, formiminoglutamate, imidazole lactate, N-acetyl-3-methylhistidine, and trans-urocanate. A histidine derivative called 3-methylhistidine was identified as the most significant metabolite associated with wooden breast and white striping in regression analysis (Fig 1; wooden breast q-value = 5.18x10^-26^, white striping q-value = 1.21x10^-09^) and also the top metabolite for classifying birds as wooden breast affected or unaffected via our linear SVM model (Table 2). Using 3-methylhistidine alone in the SVM model achieves an impressively high prediction accuracy of 82% (Table 2). 3-methylhistidine is found mainly in the contractile proteins of skeletal muscle, actin and myosin, and is one of the few amino acids that cannot be reutilized for protein synthesis [24]. After the intracellular breakdown of actin and myosin, 3-methylhistidine is released into the blood stream and excreted in urine. Its concentration in plasma and urine is used as an index of myofibrillar breakdown in skeletal muscle, though dietary intake of 3-methylhistidine in ingested muscle protein must be restricted in order to obtain accurate measurements [24]. Even though 3-methylhistidine shows high sensitivity to wooden breast status in our results, it may not have the specificity necessary to be wielded as a diagnostic marker of wooden breast or white striping due to its association with myofibrillar degeneration more generally. As such, it is possible that 3-methylhistidine may represent a “pan-myopathy” marker, differentiating any myopathy from the normal non-diseased state. Further research is needed to determine whether 3-methylhistidine is also predictive of other known broiler myopathic diseases, such as deep pectoral myopathy (green muscle disease) or nutritional myopathy related to Vitamin E/Selenium deficiency, or is unique to the wooden breast/white striping disease spectrum.

Previously, Abasht et al. [11] found elevated levels of 3-methylhistidine, histidine, and 1-methylhistidine in wooden breast affected pectoralis major compared to unaffected pectoralis major samples. Vignale et al. [25] also found higher concentrations of 3-methylhistidine, expressed as a higher fractional breakdown rate, in broiler breast muscle samples with severe white striping compared to normal samples. These findings are consistent with histological and compositional observations associated with wooden breast, specifically a decrease in protein content in the pectoralis major and a reduced number of muscle fibers [26]. It is important to note that protein breakdown rates and the associated changes to 3-methylhistidine in the breast muscle and excreta of broilers have been associated with differences in body composition and feed efficiency [27,28]. However, birds that are more susceptible to wooden breast and white striping tend to have high feed efficiency and high breast muscle yield, which are generally associated with reduced protein breakdown, or reduced 3-methylhistidine, at least in response to greater protein intake [27,28].

In humans, increased levels of 1-methylhistidine and 3-methylhistidine in urine are associated with obesity and uncontrolled diabetes mellitus [29,30]. This is consistent with previous findings from our laboratory that support similarities in the pathogenesis of broiler wooden breast and white striping and mammalian metabolic syndrome and type 2 diabetes [3,7,31], although this comparison does not hold for histidine and histamine. It has been reported that lower plasma concentrations of histidine and higher plasma concentrations of histamine are associated with obesity and type 2 diabetes [32,33], with histidine supplementation contributing to amelioration of metabolic syndrome including improvements to inflammation and oxidative stress [34]. Histidine supplementation in chickens also produces anti-oxidant benefits [35], although at high levels it can drastically reduce weight gain in growing birds [36].

### Beta-alanine and taurine metabolism

**Fig 4:**
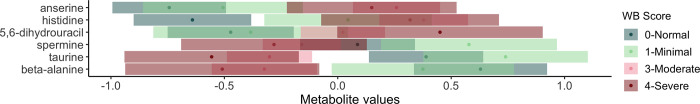
Association of wooden breast score with beta-alanine and taurine metabolism in plasma of male broiler chickens. Results of post-hoc analysis of metabolite values by wooden breast score are shown with estimated marginal means (dots) and confidence intervals (bars). Overlapping confidence intervals indicate that there is no significant difference between the two scores. To improve visualization, metabolites are listed in order of the adjusted means for a wooden breast score of 0-Normal.

One manner by which histidine supplementation improves antioxidant status is by increasing levels of the histidine-derived antioxidants (also known as histidine containing dipeptides, or HCDs) anserine and carnosine in muscle. Anserine and carnosine were previously found to be reduced in wooden breast-affected pectoralis major muscle [18], providing further evidence of altered redox homeostasis associated with the myopathy. In plasma, we found that anserine was increased in birds with greater wooden breast severity (Fig 4) although there was no significant association between wooden breast score and carnosine levels. A likely contributor to low anserine and carnosine in the pectoralis major muscle is insufficient plasma beta-alanine (Fig 4), as the rate of carnosine and anserine synthesis in skeletal muscle is limited by circulating availability of the precursor beta-alanine.

It is unclear whether reduced plasma beta-alanine in wooden breast is caused by its depletion in response to oxidative stress or by some other mechanism, but can contextualized by an investigation into the primary mechanism of HCD action in avian skeletal conducted by Dolan et al. [37] using a physiological comparison of hummingbird and chicken pectoralis major. Hummingbirds have highly oxidative and contractile pectoralis major suitable for high wing-beat frequency, sustained flight, and rapid change in flight speed and trajectory. Chicken pectoralis major is primarily glycolytic, useful for quick bursts of energy to escape danger but largely vestigial in terms of flight. Dolan et al. [37] found that hummingbird pectoralis major contained very low levels of HCDs (7.46 ± 2.6 mmol.kgDM^−1^) while chicken pectoralis major contained very high levels of HCDs (206.69 ± 17.76 mmol.kgDM^−1^). Oxidative stress response and Ca^2+^ signaling were ruled out as the major function of HCDs based on evaluation of mitochondrial content, superoxide dismutase activity, and measures of contractility. Rather, the author proposed that HCDs functioned primarily as intracellular proton buffers, protecting intramuscular pH homeostasis when hydrogen ion accumulation occurs during anaerobic metabolism [37]. This interpretation may inform our understanding of elevated ultimate pH [38] and increased expression of slow myofiber-specific isoforms [39] in wooden breast affected muscle, which has lower levels of HCDs in muscle and lower levels of the HCD precursor beta-alanine in plasma.

In humans, beta-alanine uptake into skeletal muscle cells is thought to be mediated by two transporters, proton-coupled amino acid transporter 1 (PAT1) and taurine transporter (TauT), which also regulate the uptake of another beta amino acid, taurine [39]. Taurine exerts a wide range of physiological functions, serving most notably as an antioxidant, calcium modulator, and osmoregulator [39]. In the present study, taurine was inversely related to both wooden breast and white striping score (S2 and S3 Tables), potentially due to increased uptake in skeletal muscle as Abasht et al. [11] documented elevated taurine levels in the pectoralis major muscle of wooden breast affected chickens. The importance of plasma taurine deficiency has mainly been demonstrated by taurine supplementation in animal models and clinical trials where it can prevent or mitigate various aspects of metabolic syndrome, including hyperglycemia, dyslipidemia, hypertension, oxidative stress, and inflammation [40,41]. In one study of cardiac disease in Wistar rats, a combined treatment of taurine and beta-alanine had dramatic effects on markers of oxidative stress and inflammation, increasing activity of glutathione peroxidase and superoxide dismutase by more than 175% and reducing serum levels of tumor necrosis factor (TNF)-α and interleukin-6 (IL-6) by nearly 60% [42].

### Sphingolipid metabolism

**Fig 5:**
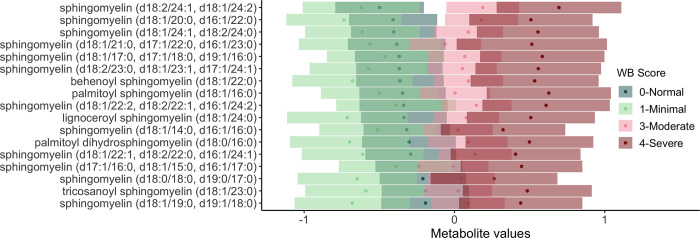
Association of wooden breast score with sphingolipid metabolism in plasma of male broiler chickens. Results of post-hoc analysis of metabolite values by wooden breast score are shown with estimated marginal means (dots) and confidence intervals (bars). Overlapping confidence intervals indicate that there is no significant difference between the two scores. To improve visualization, metabolites are listed in order of the adjusted means for a wooden breast score of 0-Normal.

Elevated levels of sphingomyelins among birds with severe wooden breast (Fig 5) and white striping may be important with regard to vascular changes and lipid accumulation associated with these myopathies. Inflammation of small- and medium-sized veins accompanied by perivenous lipid infiltration was reported by Papah et al. [9] as an early histological lesion associated with wooden breast in birds as young as 1 week post-hatch. These lesions had features in common with atherosclerosis despite being localized to veins alone, whereas atherosclerosis in birds more commonly affects arterial vessels. Human and rodent studies have found that plasma sphingomyelin levels are highly correlated with and considered a significant risk factor independent of cholesterol for coronary artery disease and subclinical atherosclerosis [43,44]. Sphingomyelin is an important component of circulating lipoproteins, and is enriched in atherogenic triglyceride-rich lipoprotein remnants because it is not degraded by plasma enzymes and instead relies on hepatic clearance methods for removal from plasma [43].

In wooden breast, there is strong evidence for increased lipoprotein metabolism in the veins of the pectoralis major, where expression of the *lipoprotein lipase* (*LPL*) gene is increased even from an early age [10,31,39]. It is possible that greater lipoprotein metabolism in veins of wooden breast affected birds results in higher levels of sphingomyelins in venous plasma, where they may contribute to perivascular lipid leakage, deposition and subsequent inflammation. Sphingomyelins comprise approximately 85% of sphingolipids in humans and serve as structural cell membrane components as well as critical signaling molecules [45]. Although sphingolipids have not been well-studied in chickens, they have been proposed to be among the most pathogenic lipids in the development of metabolic disorders related to adiposity in humans, including diabetes and cardiovascular disease [46]. Sphingomyelin release from peripheral nerve damage within affected muscle tissues cannot entirely be ruled out as a potential source for this metabolite, though no significant nerve lesions are apparent histologically in wooden breast/white striping affected tissues.

### Purine and pyrimidine metabolism

**Fig 6:**
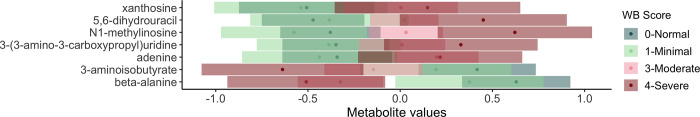
Association of wooden breast score with nucleotide metabolism in plasma of male broiler chickens. Results of post-hoc analysis of metabolite values by wooden breast score are shown with estimated marginal means (dots) and confidence intervals (bars). Overlapping confidence intervals indicate that there is no significant difference between the two scores. To improve visualization, metabolites are listed in order of the adjusted means for a wooden breast score of 0-Normal.

Wooden breast affected birds exhibited altered purine metabolism, with elevated levels of adenine, N1-methylinosine, and xanthosine (Fig 6). Metabolites involved in pyrimidine metabolism were also impacted, with wooden breast affected birds showing higher levels of 5,6-dihydrouracil and 3-(3-amino-3-carboxypropyl)uridine, but lower levels of beta-alanine and 3-aminoisobutyrate (Fig 6). Abasht et al. [11] previously identified changes to nucleotide metabolism in wooden breast affected pectoralis major muscle involving the accumulation of cytidine, thymidine, adenine, uridine, guanosine, and several nucleotide catabolites. This was believed to result from increased activity of the pentose phosphate pathway or decreased nucleotide utilization [11]. While it is unclear whether the changes to plasma nucleotide metabolism described here directly reflect alterations occurring in the pectoralis major, one metabolite requires additional scrutiny based on its connection to skeletal muscle, 3-aminoisobutyrate.

The thymine catabolite 3-aminoisobutyrate, which we found to be reduced in birds severely affected by wooden breast compared to unaffected and minimally affected birds (Fig 6), functions as a small molecule myokine that is secreted from skeletal muscle cells both at rest and in response to exercise [47], causing an increase in plasma levels of the metabolite. In mammals and rodents, 3-aminoisobutyrate is inversely correlated with cardiometabolic risk factors at least partly due to several identified regulatory mechanisms in inflammation and energy metabolism, including expression of brown adipocyte-specific genes, hepatic fatty acid oxidation, insulin release from pancreatic beta cells, and insulin sensitivity of the liver, adipose tissue, and skeletal muscle [48–50]. These effects of 3-aminoisobutyrate are at least in part mediated by activation of AMP-activated protein kinase (AMPK) and involve major metabolic transcription factors such as peroxisome proliferator-activated receptors α/δ/γ, nuclear factor kappa B (Nf-κB), and sterol regulatory element-binding protein-1c (SREBP-1c) [51]. A reduction of plasma 3-aminoisobutyrate in wooden breast affected birds may signal dysregulation of lipid and glucose metabolism, which is well-documented in wooden breast [7,31,39].

Plasma adenine shifts, with levels moderately increased in birds with a score of 3 or 4 compared to birds with a score of 0 or 1 (Fig 6), may also result from altered energy metabolism in wooden breast. In an obese diabetic mouse model, increasing plasma free fatty acids produced a dose-dependent increase in adenine nucleotides and reduction in glucose tolerance [52]. This effect was limited to the diabetic condition and was not recorded in other obese mouse models. The authors of that study identified two potential sources contributing to the increase in adenine nucleotides– their release from endothelial cells prior to apoptosis and their release from red blood cells in response to hydrogen peroxide [52]. In the wooden breast condition, this may reflect generation of reactive oxygen species.

### Carnitine and fatty acid metabolism

**Fig 7:**
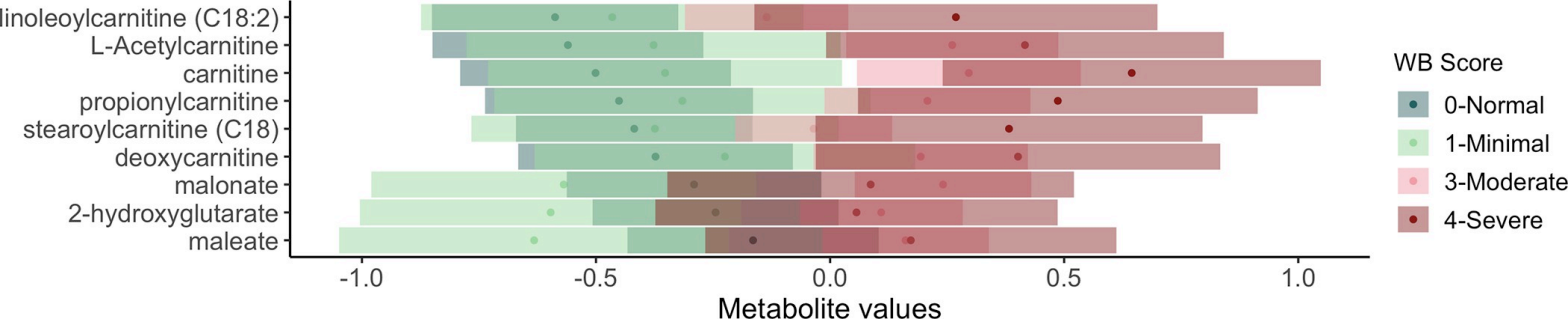
Association of wooden breast score with carnitine and fatty acid metabolism in plasma of male broiler chickens. Results of post-hoc analysis of metabolite values by wooden breast score are shown with estimated marginal means estimated marginal means (dots) and confidence intervals (bars). Overlapping confidence intervals indicate that there is no significant difference between the two scores. To improve visualization, metabolites are listed in order of the adjusted means for a wooden breast score of 0-Normal.

Carnitine is a branched amino acid that, at least in mammals, can either be absorbed from dietary intake or synthesized in the liver or kidneys from lysine and methionine. It plays a critical role in energy metabolism, especially in cardiac and skeletal muscle, via its involvement as a cofactor in the mitochondrial beta-oxidation of long-chain fatty acids along with its most abundant derivative, L-acetylcarnitine. Both carnitine and L-acetylcarnitine are increased in plasma of moderately and severely affected birds compared to unaffected birds (Fig 7). In contrast, free carnitine is reduced in the pectoralis major muscle of wooden breast affected birds and multiple long chain fatty acids are increased [11]. Together, this may indicate altered uptake of carnitine, which must be transported into skeletal muscle from plasma, either preceding or resulting from other changes in lipid metabolism that have been documented. For example, increased expression of the *LPL* gene in wooden breast- affected pectoralis major suggests an increased uptake of fatty acids from circulating lipoproteins, as *LPL* encodes the rate-limiting enzyme in lipoprotein metabolism [31,39]. Evidence supporting altered lipid metabolism in wooden breast has been demonstrated in numerous studies including a recent metabolomics study by Kong et al (2021) who reported elevated levels of 3-hydroxybutyric acid in blood plasm from 4-week-old WB affected chickens [53].

### Inositol metabolism

One of the four metabolites unique to white striping was chiro-inositol, which is reduced in birds with more severe white striping scores (S3 Table). Despite being the only metabolite in this pathway significantly affected by white striping, this compound is worth mentioning because it has been studied mainly as a component of insulin signaling and disposal of intracellular glucose. Chiro-inositol functions as part of a second messenger system for insulin, and even acts as an insulin mimetic, by activating Mg2+-dependent protein phosphatases involved in oxidative and nonoxidative glucose metabolism [54]. It was also demonstrated in several studies that progression from normal glucose tolerance to type 2 diabetes was associated with worsening chiro-inositol deficiency due to the increasing resistance to insulin [54]. Reduced plasma chiro-inositol in white striping-affected birds suggests that insulin resistance may be a component of the disease, although substantial differences in insulin signaling between mammalian and avian species complicate direct comparisons of circulating metabolites.

## Conclusion

Wooden breast and white striping incidence had the most notable association with metabolites involved in histidine metabolism and sphingolipid metabolism, potentially resulting from increased muscle protein breakdown and increased lipoprotein uptake in the pectoralis major of affected birds. Specifically, 3-methylhistidine and N-acetyl-L-aspartic acid were both identified as highly significant by mixed linear model analysis for both wooden breast and white striping and were part of the optimal subset of metabolites identified by linear support vector machine. However, their use as a potential diagnostic measure for wooden breast and white striping should be further scrutinized because 3-methylhistidine reflects muscle degradation, which is not specific to these muscle disorders. Perhaps a model based on a combination of 3-methylhistidine and N-acetyl-L-aspartic acid as well as palpation scores could provide a more specific diagnostic measure for wooden breast.

## Supporting information

S1 Table Raw values for the 615 metabolites identified and quantified by Metabolon. (XLSX)

S2 Table Results of post-hoc analysis for metabolites significantly associated (q-value < 0.05) with wooden breast score. (CSV)

S3 Table Results of post-hoc analysis for metabolites significantly associated (q-value < 0.05) with white striping score. (CSV)

S4 Table Heritability estimates for metabolites significantly associated with wooden breast score in mixed linear model analysis. Heritability was estimated from genetic marker data.
(XLSX)

S5 Table The full confusion matrices reporting the number of true positives, true negatives, false positives, and false negatives for each step of the stepwise selection support vector machine. (XLSX)

S1 File Details of sample preparation and metabolomic profiling performed by Metabolon. (DOCX)
